# Tendon healing is adversely affected by low-grade inflammation

**DOI:** 10.1186/s13018-021-02811-w

**Published:** 2021-12-04

**Authors:** Emanuele Chisari, Laura Rehak, Wasim S. Khan, Nicola Maffulli

**Affiliations:** 1 University of Catania, 95123 Catania, Italy; 2 Athena Biomedical Innovations, Florence, Italy; 3 Division of Trauma and Orthopaedic Surgery, Addenbrooke’s Hospital, University of Cambridge, Cambridge, CB2 0QQ UK; 4 Department of Musculoskeletal Disorders, School of Medicine and Surgery, University of Salerno, Salerno, Italy; 5 Clinica Ortopedica, Ospedale San Giovanni di Dio e Ruggi D’Aragona, 84131 Salerno, Italy; 6 Queen Mary University of London, Barts and the London School of Medicine and Dentistry, Centre for Sports and Exercise Medicine, Mile End Hospital, 275 Bancroft Road, London, E1 4DG UK; 7 School of Medicine, Institute of Science and Technology in Medicine, Guy Hilton Research Centre, Keele University, Thornburrow Drive, Hartshill, Stoke-on-Trent, ST4 7QB UK

**Keywords:** Inflammation, Tendinopathy, Healing, Proinflammatory cytokines

## Abstract

**Background:**

Tendinopathy is common, presents with pain and activity limitation, and is associated with a high risk of recurrence of the injury. Tendinopathy usually occurs as a results of a disrupted healing response to a primary injury where cellular and molecular pathways lead to low grade chronic inflammation.

**Main findings:**

There has been a renewed interest in investigating the role of Inflammation in the pathogenesis of tendinopathy, in particular during the initial phases of the condition where it may not be clinically evident. Understanding the early and late stages of tendon injury pathogenesis would help develop new and effective treatments addressed at targeting the inflammatory pathways.

**Conclusion:**

This review outlines the role of low-grade Inflammation in the pathogenesis of tendinopathy, stressing the role of proinflammatory cytokines, proteolytic enzymes and growth factors, and explores how Inflammation exerts a negative influence on the process of tendon healing.

## Introduction

Tendons are well recognized essential structure of the musculoskeletal system [1, 2]. While their microscopical and macroscopic structure is still debated, it is well recognized that tendons are basically made by an abundant extracellular matrix made of elongated type I collagen (70–80%) and elastic fibres (up to 2%), and a less predominant cellular component made by tenoblasts and tenocytes. Tenoblast, specialized fibroblast highly metabolically active, and mature spindle-shaped tenocytes which with lower metabolic activity [3]. In addition, at the origin and the insertion site, you can find mixed cellular elements such as chondrocytes, synovial cells, and pericytes [3]. The extracellular matrix surrounding the collagen and elastic fibre is rich in water trapped by glycosaminoglycans, proteoglycans and other small molecules [1, 2]. Both active remodelling of the matrix and its inhibition take part in the modulation of tendon homeostasis [1, 4]. The remodelling phase is attributed mostly to matrix metalloproteases, and the inhibition to their inhibitors (tissue inhibitors of metalloproteinases) [1, 4]. However, recent research showed how environmental factors such as mechanical load, inflammatory status, and other systemic conditions play an essential role within this dynamic balance [1, 4]. When an injury occurs, and the tendon is not able to heal properly, the disruption of this balance results in tendon inflammation, tendinopathy, or tendon injury, all common causes of musculoskeletal pain [5–7]. These injuries peak in ageing individuals and young athletes [5–7]. Chronic tendinopathy can cause restriction of daily life activities, and produces long-lasting physical and psychological effects [5–7].

Three distinct phases regulate the physiology of tendon healing after an acute injury: an inflammatory phase, a proliferative phase, and a remodelling phase. The inflammatory phase lasts three to seven days from the injury, and it is featured by a prevalence of inflammatory cells such as monocytes and macrophages [8]. In this phase, the inflammatory response to the injury elicit platelet activation and the formation of granulation tissue which allows cells to migrate from surrounding areas to the injured site. Then, the proliferation of these cells is part of the proliferative phase, which allows the regeneration of the tissue and the differentiation of the cells from fibroblast to tenoblasts and tenocytes. During this phase, mechanical solicitation are essential to allow correct healing [9]. Continuous, intermittent, or activity-related pain occurs during this phase, as part of the healing process [3, 4]. Ultimately, the remodelling phase starts with maturation and remodelling of ECM as objective. Overall, during this phase both cellular and extracellular components organize to resemble the final tendon structure and increase cross-linking among collagen fibres. The transverse area of the tendon, which increased during the proliferation phase, is now returning to its normal measures, while its mechanical properties improve. This last phase can take up to two years [3].

The disruption of the early inflammatory response part of the first stage of tendon healing plays an important role in the initiation of tendon pathologies [10–12]. In particular, the expression by tenocytes and other peritendinous tissues of several proinflammatory and antiinflammatory cytokines, have been linked to tendinopathy, such as tumour necrosis factor-alpha (TNF-α), IL-1β, IL-6, IL-10, VEGF, TGF-b, cyclo-oxygenase-2 (COX-2) and prostaglandin E2 (PGE2) [9, 13, 14]. Cytokines and mechanical load influence cell maturation, tissue metabolism [15–19], and gene expression [18] of healthy tendons. Although the inflammation-driven by cytokines might have a role in the healing process, its role in the development, healing, and complete resolution of tendinopathy, tendon rupture, and other inflammatory processes remains controversial [15, 20]. Mechanical loads play a key role in both the maintenance and recovery of tissue homeostasis [21]; sedentary individuals present higher levels of proinflammatory factors (e.g. TNF-a, IL-1 β and VEGF) and low levels of COL-I which lead to an increased activity of MMPs (MMP-2, -9, -13) with the onset of a low state of Inflammation with a higher risk of tendon rupture [8]. Without initial Inflammation, the healing process and the subsequent changes that characterize chronic tendinopathies (i.e. over12 weeks) cannot take place [22]. The various inflammatory mediators involved in tendon injury and tendinopathy, and the current literature on their role are discussed below.

### Interleukin family

Interleukin-1β (IL-1β) is an important proinflammatory cytokine implicated in diverse cellular functions and produced in conditions such as infection and injury [23]. IL-1β was thought to be produced only by monocytes and macrophages, but is now known to also be produced by other connective tissue cells [24]. Tenocytes produce inflammatory mediators such as COX-2, PGE2, and matrix metalloproteinase-1 (MMP-1), and these mediators can accelerate the degradation of tendon ECM impairing the mechanical properties of tendon [14].

**Fig. 1:**
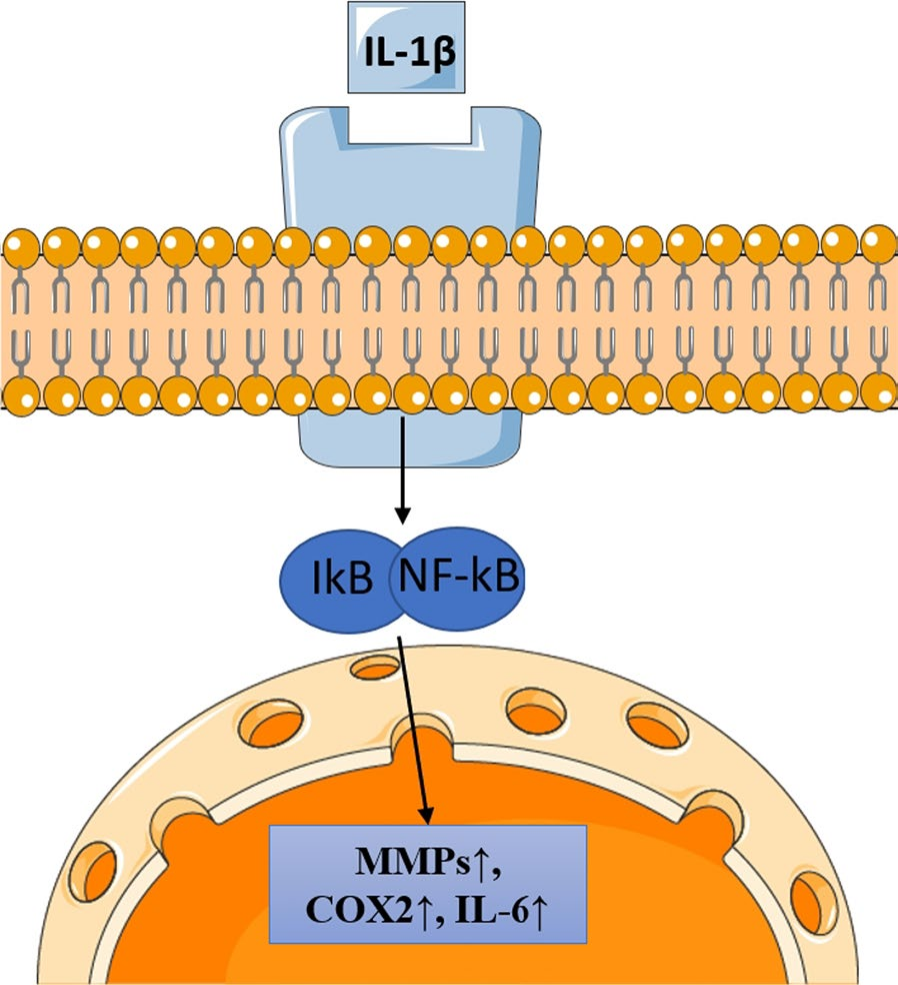
The IL-1β pathway. IL-1β interaction with its receptor causes the activation and transduction of NF-kB and IkB from cytoplasm into the nucleus where they upregulate the gene expression of acute-phase factors such as MMPs, COX2, and IL-6 triggering the Inflammation

In vitro studies have shown how the intracellular signalling pathway resulting from the interaction between IL-1β and its receptor (IL-1R) involves adapter proteins MyD88 (myeloid differentiation factor 88), IRAK (IL-1-receptor-associated-kinase) and TRAF6 (TNF-receptor associated factor-6), and leads to the activation of nuclear factor kappa-light-chain-enhancer of activated B cells (NF-κB), c-Jun N-terminal-kinase (JNK) and mitogen-activated protein kinase (MAPK) (Fig. 1) [25]. The NF- κB pathway regulates one of the strongest proinflammatory pathways studied [26, 27], influencing the expression of more than 500 different gene products linked with Inflammation, proliferation, and angiogenesis. Emerging evidence supports the role of NF-κB pathway in the maintenance of tissue homeostasis, but the role in tendinopathy and tendon inflammation remains unclear [26, 27].

In animal models of tendon injury, gene and protein expression was increased in the early stages of injury or healing for two weeks following the intervention [28–31]. Exercise also increased the gene and protein expression of IL-1β in the early stages [12, 19, 32–40]. The evidence from animal studies thus supports the role of IL-1β in the pathogenesis and progression of tendinopathy. Human studies do not however confirm these preclinical findings [22, 41–43]. IL-1β gene expression from injured or torn rotators cuff tendon tissue was decreased [41, 43–48] but remained unchanged from torn Achilles tendon tissue after repair, exercise, or tendinopathy [10, 22, 43, 49, 50]. The role of IL-1β based on human studies remains largely unknown. Cytokines belonging to the interleukin-6 (IL-6) family include IL-6 itself, as well as IL-11, oncostatin M, ciliary neurotrophic factor, leukaemia inhibitory factor, and cardiotrophin-1 [10]. IL-6 is a multifunctional Th2 produced cytokine that influences immune function and is involved in tendon healing. [16, 51].

In vitro, IL-6 induces the acute phase response and enhances the late healing phase [10], even though the molecular networks involve several other factors such as NF-κB pathway [10, 26, 27, 52]. TNF-α and IL-1β significantly promote the production of IL-6, confirming its role in Inflammation and cellular response to tissue injury [10, 52]. IL-6 is also implicated in the early phases of tendon healing, promoting the increase of COL1A1 expression in tendons [10]. In animal models of tendon injury, IL-6 gene and protein expression was increased from two hours to four weeks following the intervention [31, 33], further supporting its role in the pathogenesis of tendon disease. The effect of mechanical stress and exercise on IL-6 levels is controversial and inconclusive in animal models [17, 33, 34, 40]. The present published evidence does not support a direct pathological degeneration after IL-6 treatment on tendon cells: this underlines the involvement of other molecules to influence the tendon ECM genes and protein expression [53]. In human studies, the expression of some cytokines of the IL-6 family increased in pathological tendons [10, 17, 31, 45]. Human tendon fibroblasts secrete IL-6 when subjected to an increased level of mechanical stretching [51]. IL-6 gene and protein expression was increased in both rotator cuff and Achilles tendon tear samples [10, 17, 31, 45, 50, 54] even two weeks after surgical repair [22]. Increased protein expression was noted after prolonged exercise in healthy tendons [55] but not in Achilles tendinopathy [42].

**Fig. 2:**
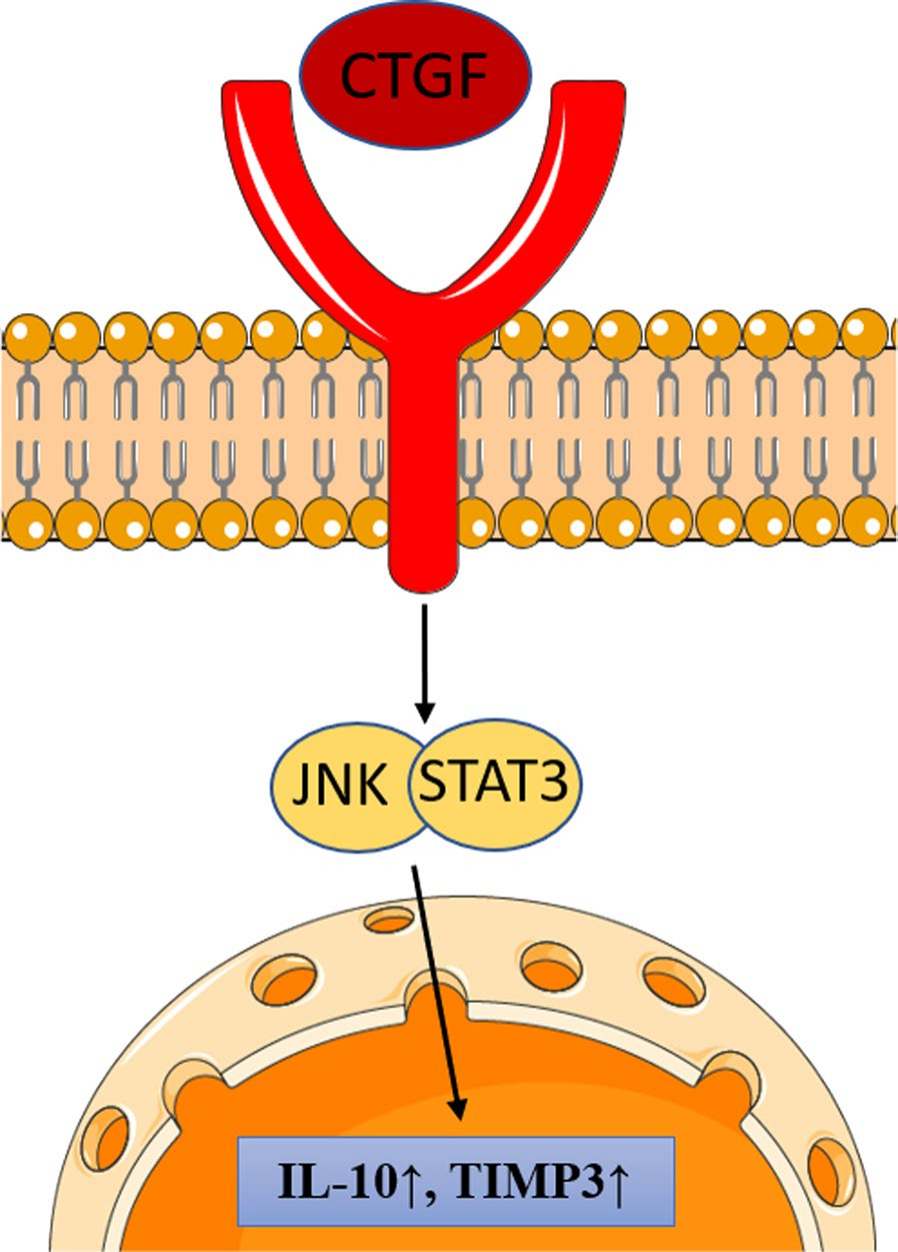
CTGF pathway. CTGF interaction with its receptor stimulates IL-10 and TIMP3 expression through the JNK/STAT3 pathway

The proinflammatory features of the cytokines mentioned above act through the NF-κB pathway. This pathway is crucial in animal models of chronic tendinopathy, where its knockout is protected against the development of the condition [56]. Also, a possible target of Interleukin family cytokines is the involvement of the extracellular matrix through a higher expression of proteins associated HMGB-1 and upregulation of NLRP3 inflammasome pathway, TLR4, TLR2, TREM-1, RAGE, ASC, Caspase-1 [57] (Fig. 2).

In vitro, IL-10 expression was only detected when tendon cells were stimulated with IL-1β and CTGF [58]. Similarly, TIMP-3 expression was only detected when tenocytes were treated with CTGF or CTGF with IL-1β [58]. IL-10 and TIMP-3 were significantly higher in CD146^+^ stem/progenitor cells than CD146^−^ tenocytes [58]. Further, signalling studies with specific inhibitors and Western blot analysis demonstrated that connective tissue growth factor-induced expression of IL-10 and TIMP-3 in CD146^+^ stem/progenitor cells are regulated by JNK/signal transducer and activator of transcription three signalling [58]. These findings suggest an antiinflammatory role of CTGF-stimulated tendon stem/progenitor cells associated with improved tendon healing. The expression of IL-10 is, however, inconsistent in clinical samples [41–43, 45] and animal injury models [31], with no effects of exercise noted in either humans or animals [33, 38–40]. Other cytokines involved in tendon healing include IL-4, IL-13, [50] IL-8 (a potent chemotactic agent and activator of neutrophils) [22, 59, 60], IL-21 and its receptor IL-21R [22, 50, 59–61]. These cytokines play a role in the early phases of Inflammation in tendon healing, and probably in early tendinopathy, with increased tenocyte proliferation, collagen, collagenase, and protease synthesis, and increased synthesis of PGE2 with the insurgence of pain [60, 61]. Besides, IL-4, IL-10, IL-13and IL-15 are involved, especially in later healing stages [50, 60].

The expression of IL-21R is affected in the first phase of tendinopathy as seen in culture after chemical challenge with TNF-α, and IL-1β [60]. IL-21R has two different binding sites: an alpha chain that links IL-21, and a gamma chain which is common to different cytokines such as IL-15, IL-7 and IL-13 [60]. High levels of IL-4R and IL-13R (except gamma chain) are found in tenocytes, and although, they are probably involved in cell proliferation, they are not involved in collagen production, suggesting that they facilitate tendon repair through their teno-proliferative effects [60, 61].

### Tumour necrosis factor-alpha (TNF-α)

Gene and protein expression of Tumour Necrosis Factor-alpha (TNF-α) do not change significantly in samples of rotator cuff and Achilles tendinopathy and tears [17, 22, 45, 62]. In animal injury models, the TNF-α gene expression was raised from two hours to nine days following the intervention and declined two weeks later [60, 63]. The protein expression lagged, increasing after four days. The effects of exercise and mechanical stimuli on TNF-α gene and protein expression in non-pathological human tendons have not been reported, and in animal models the effects were inconclusive [12, 32, 33, 37–40]. Therefore, the effects of TNF-α on tendon cells could not be ascertained [53, 64].

### M1 and M2 subsets of macrophages population

Macrophages are a type of white blood cell involved in the injury response and repair process. Emerging evidence support the existence of at multiple subsets of macrophages, namely M1 and M2 with different antigen expositions, cytokine repertoire, and action [65]. M1 polarised (classically activated) macrophages are proinflammatory, whereas M2 (alternatively activated) macrophages can limit Inflammation [65]. When tendon injury occurs, chemotactic cytokines that promote the delivery of different cell populations to the repair site are released from the tendon ECM [66, 67]. Phagocytic neutrophils and M1 macrophages are the first to arrive within 24 h of the injury, followed by a reparative shift in function which correlates with increases in M2 macrophages [3, 31].

An increasing number of studies have recently demonstrated the presence of Inflammation in the early phases of tendinopathy [68]. Compared to human tissue biopsy samples of massive rotator cuff tears, smaller tears exhibit marked inflammatory infiltration, including the presence of macrophages [46]. Tendons at different stages of tendinopathy contain different types of macrophages, virtually absent in healthy tendons [31]. Although M1 macrophages were abundant in subacute injured tendons, chronically injured tendons contained M2 macrophages [65]. This fits with our current understanding of tendinopathy, where there is Inflammation during the early stages of tendinopathy but, as the condition progresses further, Inflammation gradually subsides, and a failed healing response ensues [17, 31, 69].

The healing process in similar conditions is severely impaired and can fail to heal, as commonly seen in tendinopathy [4, 8]. There is increasing research interest in this failed healing response contributed to by both extrinsic and intrinsic factors set in an environment of low-grade Inflammation. These features, although poorly understood, are distinctive from the essential lesion of tendinopathy [4, 8], and early intervention after tendon injury should, in theory, be able to improve the overall outcome [70].

### Vascular endothelial growth factor (VEGF)

Vascular endothelial growth factor (VEGF) is involved in the healing of several tissues, including tendons. VEGF induces neo-angiogenesis and drives Inflammation by delivering inflammatory mediators to the injured zone. HIF-1a activates VEGF, and HIF-1b heterodimer under hypoxia stress conditions [59, 71]. VEGF appears to have an ambivalent role in Inflammation after tendon injury [4, 59, 60, 71, 72]. Together with other cytokines such as IL-6, IL-21R, and IL-15, VEGF has the potential to both stimulate and inhibit healing. Through TIMPs induction and the expression of MMPs, VEGF degrades the ECM in various cell types, including tenocytes, fibroblasts, chondrocytes, and endothelial cells [60, 72]. Therefore, VEGF may play a significant role in the pathogenetic processes of degenerative tendon disease and its healing [60, 72]. Also, it promotes the proliferation of tenocytes and the release of COX-2, PGE2, and prostacyclin implicated in the onset of pain in acute tendinopathy as seen with other growth factors, i.e. EGF, FGF, TGF-b and IGF-1. [4, 9, 13].

### Metalloproteinases

Metalloproteinases (MMP) are a group of proteinases involved in remodelling and healing after tendon damage [73, 74]. The MMPs involved in tendon damage are MMP-1, -2, -3, -8, -9, -13, and -14 [75, 76], which usually act through an imbalance with their inhibitors, the TIMPs [3, 75, 77]. In particular, TIMP-3 is the most involved in the inhibition of ADAMs (a disintegrin and MMP complex) and ADAMTs (ADAMs with thrombospondin motif) that appears to be crucial in the balance of the ECM remodelling [75, 76]. MMPs play a central role in matrix metabolism and pain regulation [3, 75–77]. Different MMPs target specific collagenases [4, 75, 76]; MMP-1, -8, and -13 are involved in the disruption of COL1, COL2, and COL3, and MMP-18 (collagenase 4) is directed against COL IV. MMP-2 and -9 preferentially degrade smaller fragments of tendon, while MMP-3 and -10 (stromelysins) and MMP-7 (matrilysin) are involved in the activation of other MMPs [4, 75, 76]. Some MMPs such as MMP-2, -3, and -14 also mediate the healing processes [75, 76]. An increase in net MMP activity is likely to indicate matrix degradation that may represent part of the remodelling process in wound healing [78].

MMPs, TIMPs, and ADAMTs appear as main factors regulating the ECM network remodelling, and their levels are altered during tendon healing [3, 8, 75, 77, 79]. MMP-9 and -13 mediate tissue degradation during the early phase of healing, whereas MMP-2, -3 and -14 mediate tissue degradation and later remodelling [3, 8, 75, 77, 79].

### PGE2, substance P and peroxiredoxin

COX-2 plays a significant role in Inflammation, being involved in the pathway converting arachidonic acid into prostaglandins such as PGE2 [14, 80]. PGE2 is a mediator of pain and acute Inflammation in tendons, being produced by tenocytes and other fibroblasts after an injury [80]. It is also produced after in vitro stimulation with inflammatory cytokines such as TNF-α and IL-1β, initiating MMP mediated catabolism of tendon ECM5 and the insurgence of pain [3, 77], mediating tendon inflammation [14]. Both in vivo and in vitro experimental models suggest that increased production of PGE2 may play an essential role in the development of tendinopathy [80]. For example, repetitive mechanical loading elevates the production of PGE2 in human tenocytes [9, 13, 14, 81]. Increased expression of PGE2 upregulates MMP-1 and -3 expression and inhibits collagen type I synthesis [80]. Prostaglandins exert a regulatory role in maintaining healthy bone and ECM remodelling [61]. Also, PGE2 levels typically decrease with ageing in healthy tendons as a consequence of the reduction in tendon cellularity [61]. Overall, the role of PGE2 is still controversial. Its role in healthy tendons might explain some of the ageing processes affecting tendon structural quality and quantity. Its role in tendon healing may mostly rely on its proinflammatory action and network with other factors such as substance P.

Substance P is another important pain mediator in addition to PGE and regulates the expression of these molecules and increases the degradation of matrix [4]. Substance P has several roles, including facilitating histamine release from mast cells and thus enhancing vasodilatation and extravasation of immune cells [12]. Another candidate implicated in stress-induced tendinopathy is the antioxidant peroxiredoxin that may play a role in protecting against cellular damage [3, 61, 77, 82].

### Immune-centric approach as an innovative therapy tool

The persistent low-grade Inflammation observed in chronic tendinopathy will be particularly challenging, and integrating the diverse inflammatory and parenchymal cellular injury and repair responses remains an important goal. Recent findings have highlighted new and often unexpected roles for select immune cell types in promoting a permissive local environment for adequate cell replacement and restoration of tissue integrity [83]. The next generation of regenerative therapies may evolve from typical biomaterial-, stem cell-, or growth factor-centric approaches to an immune-centric approach [63], and regenerative strategies aimed at stimulating macrophage polarization or aiming at recruiting pro-wound healing macrophage subsets should be developed. Monocytes and macrophages can exacerbate Inflammation, promote tissue repair and fibrosis, or drive regeneration marking them as a primary target when designing regenerative strategies [84]. Moreover, macrophage and regulatory T cells (Treg) emerged as potent regulators of stem cells both in physiological and in tissue repair condition [84–86].

Injured skeletal muscle recruits macrophages from circulating monocytes. These macrophages exhibit inflammatory profiles (M1) that operate phagocytosis and rapidly convert to antiinflammatory M2, which stimulates myogenesis and fibre growth [87]. Thus, macrophages can orchestrate an effective repair process by finely tuning the sequential steps of adult myogenesis, strengthening the emerging concept that Inflammation regulates stem cell homeostasis [88, 89].

In in vitro human models, monocytes entering an inflammatory environment first polarize into M1, and then switch to M2 upon microenvironmental changes [90]. The shift from M1 to M2 was confirmed by assessing quantitative gene expression and protein production for a series of cytokines, markers, and transcriptional factors involved in both monocyte differentiation and macrophage polarization. Interestingly, genes involved in inflammatory activation belong to the same biological pathways involved in the cellular processes of monocyte-to-macrophage differentiation. This establishes a transcriptional connection between monocyte activation and differentiation, Inflammation and metabolism, and confirms that the resolution of Inflammation is strictly connected to macrophage differentiation in the tissue.

Moreover, monocytes can switch M1 into M2 to drive regeneration both in myocardial injury [91] and in diabetic non-healing ulcers [92]. The imbalance of M1/M2 macrophages is linked to the severity level of knee osteoarthritis, where the ratio of M1/M2 macrophages was remarkably higher in knee OA compared with healthy control [93]. Recruited macrophages change phenotype from M1 to M2 in situ in a mouse model of rheumatoid arthritis: macrophage polarization controls progression and resolution of the condition [94]. The essential role of macrophages was also demonstrated in endochondral ossification: successful regeneration follows the switch from the initially proinflammatory M1 macrophages to the antiinflammatory M2 phenotype [95, 96].

In a rat model of Achilles tendon transaction, loading appeared to delay the switch to an M2 type of Inflammation with more Treg cells [97]. A prolonged M1 phase following loading might increase the tendon regenerate bigger, suggesting an earlier switch to M2 as a new strategy.

Overall, these data on different types of inflamed or damaged tissues show how the polarization of macrophages is a highly conserved central event in the establishment of Inflammation but also in its resolution.

## Conclusion

The prolonged state of low-grade Inflammation seen in chronic tendinopathy may act as a risk factor after an acute tendon injury, predisposing to a failed healing response that may persist despite surgery. Further studies on inflammation pathways and antiinflammatory therapies directed at specific chronic inflammation molecular targets are necessary to investigate the topic further.

Active control of the immune system is a very plausible therapeutic strategy to induce tissue regeneration. However, one of the main challenges is to target the right immune cell populations and pathways for the tissue that need to be regenerated.

## Data Availability

Under request to the corresponding author we can provide supplementary material.

## Abbreviations

ECM: Extracellular matrix
TNF-α: Tumour necrosis factor-alpha
IL: Interleukin
VEGF: Vascular endothelial factor
TGF-b: Tumour growth factor-beta
COX-2: Cyclo-oxygenase-2
PGE2: Prostaglandin E2
IRAK: IL-1-receptor-associated-kinase)
TRAF6: TNF-receptor associated factor-6
NF-κB: Nuclear factor kappa-light-chain-enhancer of activated B cells
JNK: C-Jun N-terminal-kinase
MAPK: Mitogen-activated protein kinase
